# Supplementary material for: The telemedical platform MyaLink for remote monitoring in myasthenia gravis – rationale and protocol for a proof of concept study
Source: J Neuromuscul Dis. 2024 Dec 8;13(1):86–93. doi: 10.1177/22143602241296314 (PMC13141849; doi:10.1177/22143602241296314)
Supplement: sj-docx-1-jnd-10.1177_22143602241296314 - Supplemental material for The telemedical platform MyaLink for remote monitoring in myasthenia gravis – rationale and protocol for a proof of concept study [file sj-docx-1-jnd-10.1177_22143602241296314.docx]

|  | week  0 | week 1 | week 2 | week 3 | week  3 | week  4 | week 5 | week 6 | week  6 | week 7 | week  8 | week 9 | week  9 | week 10 | week 11 | week  12 |  |
| --- | --- | --- | --- | --- | --- | --- | --- | --- | --- | --- | --- | --- | --- | --- | --- | --- | --- |
|  |  |  |  |  |  |  |  |  |  |  |  |  |  |  |  |  |  |
| **visit/system** | base-line | app | app | app | study center | app/ physician  portal | app | app | study center | app | app/ physician  portal | app | study center | app | app | end-of-study | visit inde-  pendent |
|  |  |  |  |  |  |  |  |  |  |  |  |  |  |  |  |  |  |
|  |  |  |  |  |  |  |  |  |  |  |  |  |  |  |  |  |  |
| **group** | CG, IG | IG | IG | IG | IGQ+ | IG | IG | IG | IGQ+ | IG | IG | IG | IGQ+ | IG | IG | CG, IG | CG, IG |
| inclusion, exclusion criteria | x |  |  |  |  |  |  |  |  |  |  |  |  |  |  |  |  |
| demographics | x |  |  |  |  |  |  |  |  |  |  |  |  |  |  |  |  |
| MGFA status | x |  |  |  |  |  |  |  |  |  |  |  |  |  |  | x |  |
| MG history | x |  |  |  |  |  |  |  |  |  |  |  |  |  |  |  |  |
| medication | x |  |  |  |  |  |  |  |  |  |  |  |  |  |  | x |  |
| hospitalizations | x |  |  |  |  |  |  |  |  |  |  |  |  |  |  | x |  |
| care-related questions | x |  |  |  |  |  |  |  |  |  |  |  |  |  |  | x |  |
| exacerbations |  |  |  |  |  |  |  |  |  |  |  |  |  |  |  | x |  |
| MGFA-PIS |  |  |  |  |  |  |  |  |  |  |  |  |  |  |  | x |  |
| wearables |  | continuous assessment | | | | | | | | | | | | | | |  |
| spirometry  (FCV) |  | x | x | x | x | x | x | x | x | x | x | x | x | x | x | x | x* |
|  |  |  |  |  |  |  |  |  |  |  |  |  |  |  |  |  |  |
| single breath count test | x | x | x | x | x | x | x | x | x | x | x | x | x | x | x | x |  |
| QMG | x |  |  |  | x |  |  |  | x |  |  |  | x |  |  | x | x* |
| **PROMs** |  |  |  |  |  |  |  |  |  |  |  |  |  |  |  |  |  |
| MG-ADL | x | x | x | x |  | x | x | x |  | x | x | x |  | x | x | x |  |
| MG-QoL15r | x | x | x | x |  | x | x | x |  | x | x | x |  | x | x | x |  |
| SSQ | x | x | x | x |  | x | x | x |  | x | x | x |  | x | x | x |  |
| PASS | x |  |  |  |  | x |  |  |  |  | x |  |  |  |  | x |  |
| CFS | x |  |  |  |  | x |  |  |  |  | x |  |  |  |  |  |  |
| HADS | x |  |  |  |  | x |  |  |  |  | x |  |  |  |  |  |  |
| EQ-5D-5L | x |  |  |  |  |  |  |  |  |  |  |  |  |  |  | x |  |
| telemedical check up |  |  |  |  |  | x |  |  |  |  | x |  |  |  |  |  |  |
| communication patterns |  |  |  |  |  | x |  |  |  |  | x |  |  |  |  | x |  |
| usability questionnaires |  |  |  |  |  |  |  |  |  |  |  |  |  |  | x |  |  |

**Supplementary material**

Supplementary Table 1: Visit plan of randomized controlled study (N=45) with a study duration over 12 weeks.

x*: additional measurements or clinical data independent from study protocol was possible (e.g., when patient was hospitalized)
